# Phytochemical Profile, Antioxidant, Antimicrobial and Cytoprotective Effects of Cornelian Cherry (*Cornus mas* L.) Fruit Extracts

**DOI:** 10.3390/ph16030420

**Published:** 2023-03-09

**Authors:** Mara Aurori, Mihaela Niculae, Daniela Hanganu, Emoke Pall, Mihai Cenariu, Dan Cristian Vodnar, Andrea Bunea, Nicodim Fiţ, Sanda Andrei

**Affiliations:** 1 Department of Preclinical Sciences, Faculty of Veterinary Medicine, University of Agricultural Sciences and Veterinary Medicine Cluj-Napoca, 400372 Cluj-Napoca, Romania; 2 Department of Clinical Sciences, Faculty of Veterinary Medicine, University of Agricultural Sciences and Veterinary Medicine Cluj-Napoca, 400372 Cluj-Napoca, Romania; 3 Department of Pharmacognosy, Faculty of Pharmacy, University of Medicine and Pharmacy “Iuliu Haţieganu”, 400372 Cluj-Napoca, Romania; 4 Department of Food Science, Faculty of Food Science and Technology, University of Agricultural Sciences and Veterinary Medicine Cluj-Napoca, 400372 Cluj-Napoca, Romania; 5 Department of Biochemistry, Faculty of Animal Science and Biotechnology, University of Agricultural Sciences and Veterinary Medicine Cluj-Napoca, 400372 Cluj-Napoca, Romania; 6 Department of Microbiology, Faculty of Veterinary Medicine, University of Agricultural Sciences and Veterinary Medicine Cluj-Napoca, 400372 Cluj-Napoca, Romania

**Keywords:** *Cornus mas* L. fruits, phenolic compounds, carotenoids, antioxidant capacity, antimicrobial activity, cytotoxicity, renal cell injury

## Abstract

*Cornus mas* L. is characterized by an increased quantity of bioactive compounds, namely polyphenols, monoterpenes, organic acids, vitamin C and lipophilic compounds such as carotenoids, being anciently used in the treatment of various diseases. This paper’s objectives were to characterize the phytochemical profile of *Cornus mas* L. fruits and to evaluate the in vitro antioxidant, antimicrobial and cytoprotective effects on renal cells exposed to gentamicin. As such, two ethanolic extracts were obtained. The resulting extracts were used to assess the total polyphenols, flavonoids and carotenoids through spectral and chromatographic methods. The antioxidant capacity was assessed using DPPH and FRAP assays. Due to the high content of phenolic compounds analyzed in fruits and the results obtained regarding antioxidant capacity, we decided to further use the ethanolic extract to investigate the in vitro antimicrobial and cytoprotective effects on renal cells stressed with gentamicin. The antimicrobial activity was assessed using agar well diffusion and broth microdilution methods, with great results regarding *Pseudomonas aeruginosa*. The cytotoxic activity was assessed using MTT and Annexin-V assays. According to the findings, extract-treated cells had a higher cell viability. However, at high concentrations, viability was shown to decline, most likely due to the extract and gentamicin’s additive effects.

## 1. Introduction

Recently, it has been considered that nutrition plays a crucial role in human health. Therefore, interest in living a healthy way of life exists, by having a healthy diet and consuming food supplements [1,2]. Most people use plant-derived drugs as the first line in the treatment of various diseases [3]. There are studies that have proven the effectiveness of plant-derived drugs against certain illnesses, namely degenerative diseases, atherosclerosis, diabetes, gastrointestinal disorders and cancer, outlining the importance of alternative medicine compared to synthetic drugs [4,5,6,7,8,9,10,11,12]. Thus, the search for biologically active compounds from plants implies a new challenge, with the aim of obtaining new commercial products. It might be considered such plants to be berries, known for their increased quantity of sugars and phytochemical compounds [13]. Specifically, cranberries, blueberries, strawberries or cherries, with a particular interest in cornelian cherry, where recent studies suggest that it has great potential for alternative medicine applicabilities [14,15]. 

European cornelian cherry (*Cornus mas* L.), widely known as dogwood, is a flowering medicinal plant that belongs to the *Cornaceae* family. It arises from the foothills of the Caucasus Mountains and spreads over southeastern Europe, more specifically over Turkey, Bulgaria, Romania and Italy [16,17,18].

Cornelian cherry is a deciduous shrub with a height ranging from 5 to 12 m. It has dark brown branches, greenish twigs and early-blooming yellow flowers. The fruits have an oval form that resembles an olive, are firm to the touch, and only have one stone within. They bloom from September to October [16,18]. Their color is initially yellow, but as the fruits ripen, they turn crimson. Ripen fruits are edible, having an acidic and sweet flavor. Depending on the plant genotype and culture conditions, the fruit of *Cornus mas* L. weighs between 1 and 10 g and 5.7–11% of the weight is made up of the stone [17,19]. *Cornus mas* L. thrives in unaltered natural circumstances without pesticides, being therefore appropriate for cultivation in accordance with the principles of organic agriculture [20]. 

It was established by previous investigations that *Cornus mas* L. is a rich source of biologically active organic compounds. Therefore, from a phytochemical perspective, cornelian cherry holds an increased quantity of polyphenols, monoterpenes, organic acids and vitamin C. Anthocyanins, cinnamic acids, flavonoids, benzoic acids, catechins and tannins are the primary polyphenols, having the highest preponderance of 37.76%. Organic acids and monoterpenes, particularly iridoids, are present in roughly equal amounts (25.9% and 26.3%, respectively). The main organic acids identified in *Cornus mas* L. fruits include tartaric acid, malic acid, citric acid, gallic acid, loganin and chlorogenic acid. Moreover, these fruits contain 10.7% vitamin C. However, the amount of nutrients might differ based on a number of variables, including the geographical region, the cultivar and the level of ripeness of the fruit [17,19,21,22].

Cornelian cherry has traditionally been used both for food consumption and as an ornamental plant. It is cultivated in some countries for the production of marmalade, compote, syrup, juice, yogurt, liqueur and wine [16,23,24]. In addition, *Cornus mas* L. fruits are also utilized in the cosmetic industry [21]. For ornamental purposes, cornelian cherry trees are planted and grown in households, public gardens and parks due to its lovely yellow blooms [25]. 

In folk medicine, cornelian cherry fruits have historically been used to cure a variety of illnesses. The Caucasus, Central Asia, Turkey, Azerbaijan, Iran, Greece and Slovakia were the main regions where they were employed. Thus, the ripe fruits were used to treat sore throats, gastrointestinal disorders, measles, chicken pox, anemia, rickets, fever, inflammation, skin disorders, tuberculosis, malaria, cancer, thermic fever, wounds, liver diseases such as hepatitis A and kidney diseases such as pyelonephritis and renal calculi [16,21]. In recent years, the effects of *Cornus mas* L. fruits have been the subject of numerous studies, which have revealed that they have anti-inflammatory, antibacterial, antioxidant, antidiabetic, antiatherosclerotic, antiproliferative, antiparasitic, antihyperlipidemic, nephroprotective, hepatoprotective and neuroprotective effects [18,26,27,28,29,30,31,32,33,34,35,36]. 

Due to the fact that *Cornus mas* L. fruits contain a number of significant active components and due to recent epidemiological studies that have suggested that some plant extracts may be advantageous for their capacity to prevent the onset or slow the evolution of a number of illnesses, we believe it is vital to thoroughly investigate the profile of biologically active organic compounds and their effects at a cellular level. Therefore, the current study intends to identify and quantify the biologically active chemical compounds from cornelian cherry fruits as well as investigate the in vitro antioxidant, antimicrobial and cytoprotective effects on primary renal cells stressed with gentamicin, the latter of which has received very little research and may help in the development of new therapeutic agents to prevent kidney damage. 

## 2. Results

### 2.1. Total Content of Polyphenols, Flavonoids and Carotenoids of Cornus mas L. Fruit extracts

Initially, one of our objectives was to detect and evaluate the bioactive constituents of cornelian cherry cultivars from Cluj county, Romania, which may have antioxidant, antimicrobial and cytoprotective properties. 

The results regarding total phenolic content, total flavonoids and total carotenoid pigments are provided in Table 1. 

As such, the analysis of the ethanolic extracts of *Cornus mas* L. fruits revealed that the total polyphenolic content was 0.872 ± 0.0035 mg GAE/mL and the total flavonoid content was 139.14 ± 2.10 µg/mL. Total carotenoids were found to be 3.8 ± 0.0002 µg/g, resulting from the second type of extracts (ethyl ether re-extraction of the ethanolic extracts of *Cornus mas* L. fruits).

### 2.2. Identification of Phenolic Compounds by HPLC-DAD-ESI-MS

**Figure 1:**
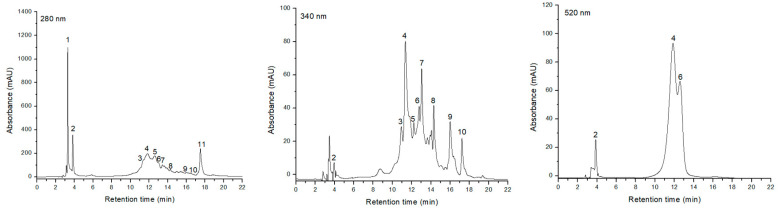
HPLC chromatograms of phenolic compounds of *Cornus mas* L. extract. Identification and quantification of phenolic acids at λ = 280 nm, flavonoids at λ = 340 nm and anthocyanins at λ = 520 nm. Peaks’ numbers refer to the compounds listed in Table 2.

The results regarding HPLC-DAD-ESI+ profile of phenolic composition are shown in Figure 1 and Table 2. The retention time, UV-Vis spectrum and each individual peak’s spectral mass were compared to published data in order to identify the phenolic compounds.

As a result, 13 compounds belonging to four different classes were identified. Thus, phenolic acids were represented by gallic acid glucoside, chlorogenic acid and caffeic acid. The class of anthocyanins was represented by coumaroyl-glucoside, Cy 3-O-galactoside, Cy 3-O-robinobioside, Pg 3-O-galactoside and Pg 3-O-robinobioside, being the most numerous class. Loganin and sweroside were also discovered, being part of the iridoid class, while rutin, K 3-O-galactoside and a procyanidin dimer were associated with the flavonol class.

Using the calculation method mentioned in the Materials and Methods section, the highest concentration of GAE was found in gallic acid glucoside (248.51 μg/mL), a hydroxybenzoic acid, followed by a procyanidin dimer (195.82 μg/mL) and loganin (111.47 μg/mL), with Cy3-O-(coumaroyl-glucoside) having the lowest concentration (6.43 μg/mL). In addition, the total phenolic content was 0.847 mg GAE/mL of extract. This result was comparable to the spectrophotometric assay of these compounds by the Folin–Ciocalteu method (0.847 mg GAE/mL vs. 0.872 mg GAE/mL).

### 2.3. Antioxidant Capacity

Using the Radical Scavenging Activity (DPPH) assay to measure the antioxidant capacity, a final IC_50_ value of 0.466 mg/mL was obtained. Simultaneously, by employing the Ferric Reducing Antioxidant Power (FRAP) method, a value of 23.09 was yielded, which was expressed as µmol Trolox/mL extract.

### 2.4. Antimicrobial Activity

The results of *Cornus mas* L. extract in vitro antimicrobial properties assessment are presented in Table 3 (zone of inhibition) and Table 4 (MIC index).

*Cornus mas* L. extract displayed in vitro antimicrobial potential against all tested bacterial strains (Table 3). The highest effect was recorded towards *Enterococcus faecalis* and *Pseudomonas aeruginosa* with diameters of the inhibition zone of 22.33 ± 0.47 mm. These results are particularly of interest since both bacteria are well known for their elevated level of antimicrobial resistance. In fact, compared to positive controls (amoxicillin–clavulanic acid for *Enterococcus faecalis* and gentamicin for *Pseudomonas aeruginosa*), the inhibition zone diameters were found significantly higher (*p* < 0.05). Further evaluation towards clinical strains isolated from human and animal cases is intended.

The extract presented inhibitory activity also against *Staphylococcus aureus* reference strains; as expected, the values were significantly higher (*p* < 0.05) in the case of MSSA compared to MRSA. The diameters of the inhibition zones were comparable to those obtained for gentamicin (*p* > 0.05) but significantly lower (*p* < 0.05) than those of amoxicillin–clavulanic acid.

The lowest inhibitory effect was recorded towards *Bacillus cereus* (inhibition zone of 17 ± 0.00 mm).

The MIC and MBC values obtained using a broth microdilution method are presented in Table 4. Based on the resulting MIC index, the extract was found to exhibit bactericidal activity against all tested bacterial strains (MBC/MIC ≤ 4).

### 2.5. Cytoprotective Activity of Cornus mas L. Extract in Gentamicin-Induced Nephrotoxicity on Primary Mice Renal Epithelial Cells

#### 2.5.1. Evaluation of Cell Viability by MTT Assay

MTT was used to assess the cytoprotective activity of the *Cornus mas* L. extract. The culture cells were initially divided into the following four groups: Control (C) group, which contained untreated cells; Gentamicin (G) group, which received antibiotic treatment in three different concentrations (G1 = 100 μg/μL, G2 = 150 μg/μL and G3 = 200 μg/μL); Cornus mas (CM) group, which received extract treatment in three distinct amounts (CM1 = 130 μg/μL, CM2 = 195 μg/μL and CM3 = 260 μg/μL) and Cornus mas + Gentamicin (GCM) group, which received treatment with a combination of both.

**Figure 2:**
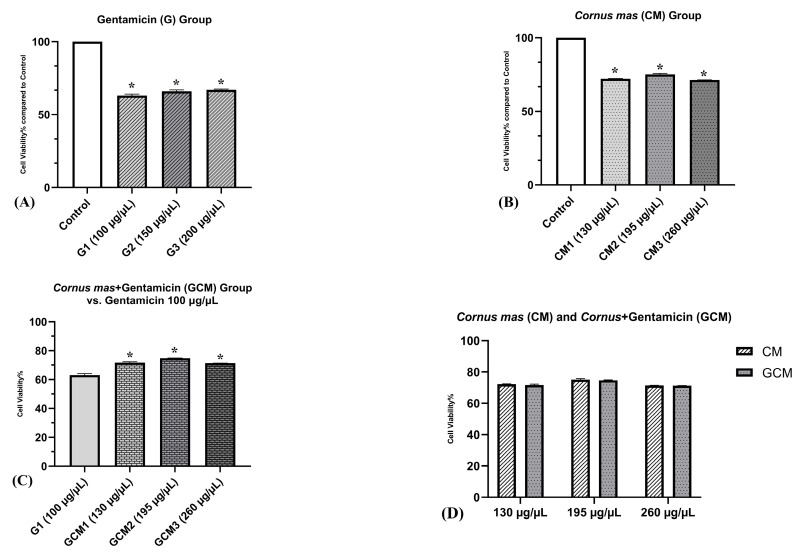
MTT test results. (**A**) Cytotoxic effect of Gentamicin in three different concentrations (G1-G3) on renal epithelial cells (one-way ANOVA; * *p* < 0.0001 vs. control). (**B**) Cytotoxic effect of *Cornus mas* fruit extract in three different concentrations (CM1-CM3) on renal epithelial cells (one-way ANOVA; * *p* < 0.0001 vs. control). (**C**) Cytotoxic effect of *Cornus mas* + Gentamicin in three different concentrations (GCM1-GCM3) on renal epithelial cells (one-way ANOVA; * *p* < 0.0001 vs. gentamicin 100 μg/μL). (**D**) Cytotoxic effect of *Cornus mas* fruit extract compared to *Cornus mas* + Gentamicin on renal epithelial cells (paired *t*-test; *p* > 0.05). The results indicate mean ± SD of triplicate measurements.

According to the results of the MTT assay, the G group’s cell viability was significantly lower than the C group’s at all concentrations (*p* < 0.05). The first concentration of gentamicin, which had the lowest viability, was found to be the most nephrotoxic, registering a percentage of 63.03%. Thus, this concentration was considered for future evaluations. Additionally, the CM group showed a statistically significant decline in cell viability in comparison to the C group, at all concentrations (*p* < 0.05). It was noticeable that the highest concentration (260 μg/μL) had the lowest viability (71.36%). Nevertheless, compared to the gentamicin-treated cells, a greater viability was observed at all concentrations. Lastly, in the GCM group, it was possible to observe a decrease in cell viability as the extract’s concentration increased. This was likely caused by the extract and gentamicin’s combined cytotoxic effects at high doses. Moreover, the cell viability in this group was higher in comparison to the cells treated with gentamicin alone (*p* < 0.05). However, the cell viability was similar to that obtained in cells treated just with *Cornus mas* L. extract, there being no statistical difference between the two groups at all concentrations (*p* > 0.05). Figure 2 displays these findings graphically. 

#### 2.5.2. Evaluation of Cell Apoptotic Rate by Annexin-V FITC Assay

Annexin-V FITC was the following test used in measuring the cytoprotective effect of *Cornus mas* L. extract in gentamicin kidney injury. Apoptotic measurements were performed on identical cell groups as presented in the MTT assay (C, G, CM1-CM3, GCM1-GCM3). Following fluorochrome staining and the cells’ affinity for PI and Annexin-V, flow-cytometry results were revealed. As such, in comparison to the C group, a statistically significant decrease in cell viability was observed in the G group (63.83% vs. 99.9%; *p* < 0.0001) and CM group (77.4%, 80.9%, 70.1% vs. 99.9%; *p* < 0.0001). It was noticeable that the second concentration of the CM group registered the highest viability, whereas the last concentration recorded the lowest level of viable cells. These results can be connected with those obtained in the MTT assay because the percentage of viable cells was shown to decline at high concentrations. Furthermore, by comparing the GCM group to the CM group, there was no statistical difference in cell viability at 130 µg/µL and 195 µg/µL concentrations (77.1%, 80.5% vs. 77.4%, 80.9%; *p* > 0.05), manifesting a protective effect in a manner similar to the MTT assay. Importantly, these two concentrations had a significantly higher percentage of viable cells when compared to the G group (77.1%, 80.5% vs. 63.83%; *p* < 0.01), outlining the protective effect of *Cornus mas* L. extract. In contrast, a significant decline in cell viability was observed at the highest concentration (260 µg/µL), compared to the CM group (62.1% vs. 70.1%; *p* < 0.05). Moreover, the percentage of viable cells of this concentration was significantly lower compared to the percentage of gentamicin-treated cells (62.1% vs. 63.83%; *p* < 0.05), probably due to the cumulative cytotoxic effect of the extract at high concentrations with that of gentamicin. 

When analyzing early apoptosis, an increase in the percentage of early apoptotic cells was observed in the G group (1.8% vs. control; *p* < 0.0001) and CM group (0.4%, 1.1%, 0.9% vs. control; *p* < 0.0001). In comparison to the CM group, the GCM group registered a similar increase in early apoptosis at the first two concentrations, namely 130 µg/µL and 195 µg/µL (1.3%, 1.1% vs. 0.4%, 1.1%; *p* > 0.05). Moreover, no significant difference was observed when compared to the G group (vs. 1.8%; *p* > 0.05). However, there was a drastic increase in the amount of early apoptotic cells at the highest concentration (260 µg/µL) of this group in comparison to the CM group (11.6% vs. 0.9%; *p* < 0.05) and the G group (vs. 1.8%; *p* < 0.01). As such, this concentration registered the highest percentage of apoptotic cells. 

Regarding tardive apoptosis, the G group registered a greater percentage of late apoptotic cells when compared to the C group (3.5% vs. 0%; *p* < 0.01). In addition, the CM group recorded a significantly higher number of tardive apoptotic cells in comparison to control (1.8%, 2.4%, 3.7% vs. 0%; *p* < 0.05). Furthermore, by analyzing the GCM group, a significantly greater decrease in these cells was observed at 130 µg/µL and 195 µg/µL concentrations, in comparison to the G group (0.2%, 1% vs. 3.5%; *p* < 0.0001). The first concentration (130 µg/µL) also recorded a significantly lower percentage of tardive apoptosis compared to the CM group (0.2% vs. 1.8%; *p* < 0.0001), whereas there was no significant difference between the GCM and CM groups at the second concentration, namely 195 µg/µL (1% vs. 2.4%; *p* > 0.05). However, the highest concentration of the GCM group recorded the greatest level of late apoptotic cells, namely 7.4%. This was significantly increased when compared to the CM group (*p* < 0.01) and G group (*p* < 0.0001).

**Figure 3:**
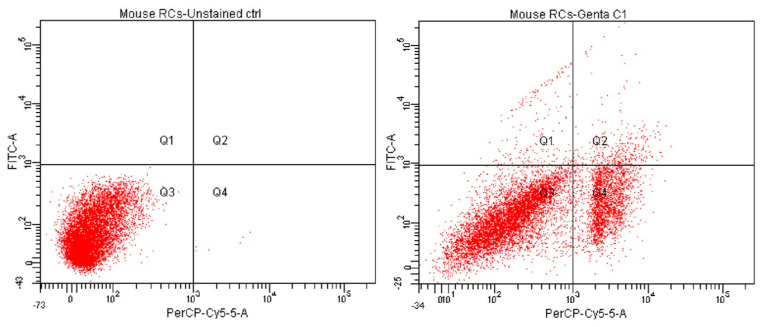
Flow cytometry analysis of cell viability of untreated and Gentamicin–treated (100 µg/µL) renal epithelial cells (Q1 = early apoptosis; Q2 = tardive apoptosis; Q3 = viable; Q4 = necrosis). The results indicate mean ± SD of triplicate measurements.

**Figure 4:**
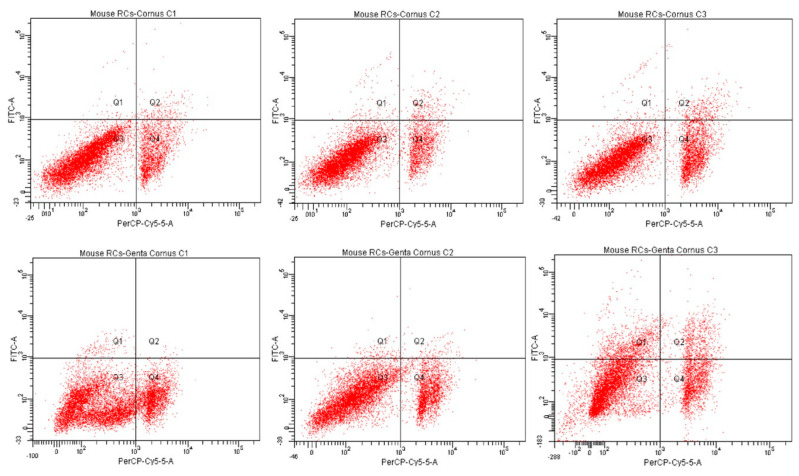
Flow cytometry analysis of cell viability of *Cornus mas*–treated (CM1–CM3) and *Cornus mas* + Gentamicin–treated (GCM1–GCM3) renal epithelial cells (Q1 = early apoptosis; Q2 = tardive apoptosis; Q3 = viable; Q4 = necrosis). The results indicate mean ± SD of triplicate measurements.

**Figure 5:**
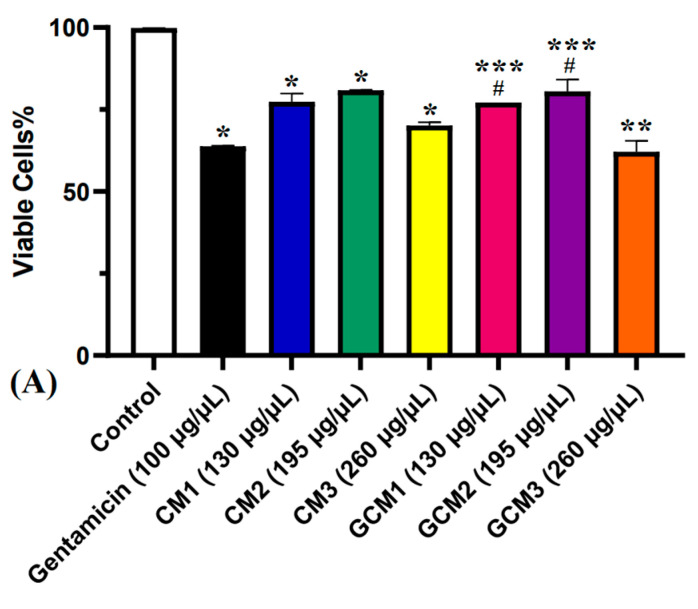

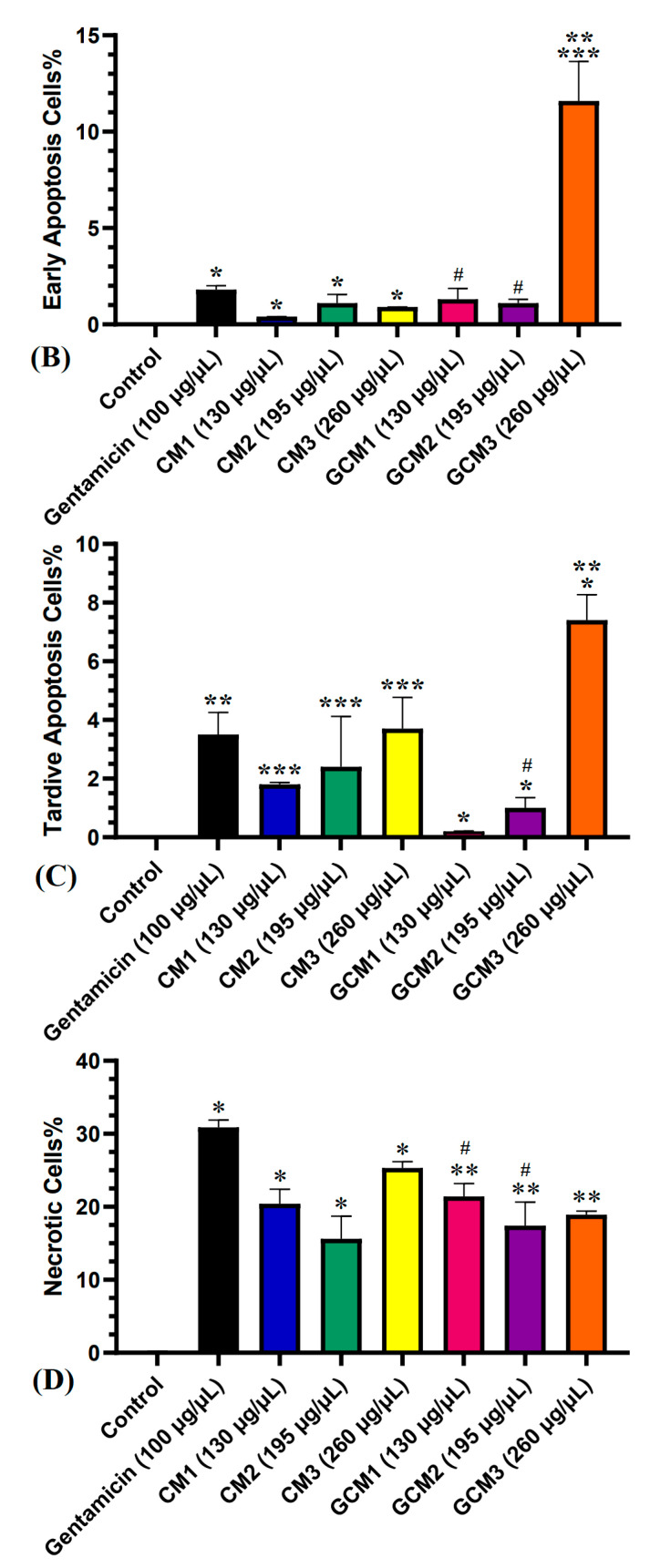
Flow cytometry analysis of (**A**) viable cells; * *p* < 0.0001 (vs. control), ** *p* < 0.05 (vs. gentamicin and CM3), *** *p* < 0.01 (vs. gentamicin), # *p* > 0.1 (vs. CM1 and CM2); (**B**) early apoptotic cells; * *p* < 0.0001 (vs. control), ** *p* < 0.01 (vs. gentamicin), *** *p* < 0.05 (vs. CM3), # *p* > 0.1 (vs. gentamicin, CM1 and CM2); (**C**) tardive apoptotic cells; * *p* < 0.0001 (vs. gentamicin and CM1), ** *p* < 0.01 (vs. control and CM3), *** *p* < 0.05 (vs. control), # *p* > 0.05 (vs. CM2); (**D**) necrotic cells; * *p* < 0.0001 (vs. control), ** *p* < 0.001 (vs. gentamicin and CM3), # *p* > 0.1 (vs. CM1 and CM2). Statistics performed utilizing one-way ANOVA, paired and unpaired *t*-tests. The results indicate mean ± SD of triplicate measurements.

Overall, necrotic cells registered a higher number in all groups when compared to early and late apoptosis. Thus, in comparison to control, there was a drastic increase in the percentage of necrotic cells in the G group (30.87% vs. 0.1%; *p* < 0.0001) and CM group (20.4%, 15.6%, 25.3% vs. 0.1%; *p* < 0.0001). The second concentration of the CM group recorded the lowest amount of necrotic cells, while the last concentration registered the highest percentage of necrosis, following the G group. Importantly, it was noticeable that the GCM group had a significantly lower percentage of necrosis in comparison to the G group (21.4%, 17.4%, 18.9% vs. 30.87%; *p* < 0.001), outlining again the importance of *Cornus mas* L. extract against gentamicin stress. Figure 3, Figure 4 and Figure 5 show a detailed replication of these outcomes. 

## 3. Discussions 

Phytochemical profile of *Cornus mas* L. is presented in this paper by assessing the total phenolic, flavonoid, and carotene content of the extracts. As a result, values of 0.872 ± 0.0035 mg GAE/mL for total polyphenols, 139.14 ± 2.100 μg/mL for flavonoids and 3.8 ± 0.0002 μg/g for total carotenoids were obtained. According to the literature, the results regarding total polyphenols and total flavonoids are similar or slightly lower than those previously reported [37,38]. Similar to these findings, the total carotenoid content was comparable to or rather decreased than earlier published data [39,40]. However, it is generally accepted that cornelian cherry fruits contain a reduced quantity of carotenoid pigments. On the other hand, when compared to other plant components, the leaves of cornelian cherry were discovered to be incredibly rich in carotenoids, especially β-carotene [41]. In addition, a more detailed characterization of the *Cornus mas* L. extract was assessed by HPLC-DAD-ESI-MS analysis. This resulted in the identification of 13 compounds belonging to four phenolic classes. Gallic acid glucoside had the highest concentration (248.51 μg GAE/mL), while Cy3-O-(coumaroyl-glucoside) had the lowest concentration (6.43 μg GAE/mL). According to Klymenko et al. [26], loganic acid had the highest concentration, ranging from 772 to 2390 μg GAE/g, while gallic acid was not identified in this study. Another research investigated the phytochemical profile of cornelian cherry fruit extract, revealing that the same compound had the greatest concentration, namely loganic acid (12913.51 μg/g). Gallic acid was identified only in yellow *Cornus mas* L. fruits, in a quantity of 263.59 μg/g, being similar to the quantity obtained in our study [42]. The composition of these compounds varies based on a wide range of parameters, including geographical region, genotype, cultivar and the degree of fruit ripening. Thus, it should be emphasized that these differences can be attributed to these factors. 

Regarding the antioxidant activity, we obtained a DPPH value of 0.466 mg/mL. In accordance with earlier studies, the result is slightly lower than those previously reported. Thus, Esroy et al. [43] reported DPPH ranging from 1.06 to 1.83 mg/mL and Celep et al. [44] registered a value of 0.72 mg/mL. In terms of the FRAP test results, we reported a value of 23.09 μmol Trolox/mL extract. This result is more elevated than those previously published in the literature. Klymenko et al. [26] registered FRAP ranging from 8.45 to 19.34 μmol Trolox/g, while Moldovan et al. [45] yielded a value of 0.628 μmol Trolox/g. Therefore, based on the previously mentioned factors, these variations can be linked to the different concentrations of phenolic compounds identified in cornelian cherry fruits. In conclusion, it should be noted that *Cornus mas* L. fruit extract exhibits in vitro antioxidant activity. 

By evaluating the antimicrobial activity, *Cornus mas* L. extract exerted antimicrobial activity against all tested strains, *Pseudomonas aeruginosa* and *Enterococcus faecalis* being the targets of the strongest effect. Moreover, the *Cornus mas* L. extract showed a significantly stronger antimicrobial potential towards these two bacterial strains when compared to amoxicillin–clavulanic acid and gentamicin. These results were compared to previously reported studies. Thus, Kyriakopoulos and Dinda [46] demonstrated the inhibitory effect of *Cornus mas* L. fresh fruits against the strains of *Staphylococcus aureus* and *Pseudomonas aeruginosa*. Another previous study investigated the antimicrobial potential of cornelian cherry fruits against four reference strains. Compared to our study, the diameters of the inhibition zone were significantly reduced for MSSA, *Pseudomonas aeruginosa* and *Escherichia coli*, being 8 mm for all strains [47]. Therefore, to the best of our knowledge, this is the first study examining the antimicrobial activity of *Cornus mas* L. fruit extract against MRSA, which may be especially intriguing for upcoming research on antibiotic resistance. Additionally, the *Cornus mas* L. fruit extract’s inhibition zone against *Pseudomonas aeruginosa* in this study had the largest diameter of any published data, strengthening the fruits’ antimicrobial characteristics.

Nephrotoxicity is one of the well-known adverse effects of gentamicin. The tubular impact of this antibiotic, which can range from a simple loss of the brush boundary in epithelial cells to a clear tubular necrosis, is a key component of its nephrotoxicity. One important factor in gentamicin’s tubular damage is oxidative stress. Thus, it has been demonstrated that diverse antioxidants counteract the harmful effects of oxidative stress. A variety of plants contain antioxidant properties due to their increased quantity in bioactive constituents, such as phenolic compounds [48]. Water spinach, red grape, garlic and green tea are a few examples of such antioxidant-enriched plants that have been shown to have an antioxidant effect in gentamicin-induced kidney damage [49,50,51]. As previously mentioned, *Cornus mas* L. is also a plant with a high concentration of phenolic compounds that have antioxidant properties. The constituents from *Cornus mas* L. fruits may have a protective impact on gentamicin-induced nephrotoxicity, a potential use for this plant for which there has been minimal previous research. As such, we believed it was vital to perform a more thorough investigation of the cytoprotective effect of these fruits on renal epithelial cells exposed to gentamicin. This effect was analyzed through cell viability assays, namely MTT and Annexin-V FITC. 

By applying the MTT assay, it was revealed that the extract-treated cells and gentamicin-treated cells both had a decrease in cell viability when compared to control (*p* < 0.0001). Nevertheless, the extract-treated cells had a higher cell viability than gentamicin-treated cells. Interestingly, it was observed that the cell viability was shown to decline as the extract’s concentration increased, with the greatest concentration registering the lowest viability. This outcome might be explained by the pro-oxidant properties of polyphenols in high quantities. Importantly, when compared to gentamicin-treated cells, there was a significant increase in cell viability in the group of cells treated with gentamicin and *Cornus mas* L. extract (*p* < 0.0001). In addition, a similar increase in viability to the extract-treated cells was observed in the group of cells that received both an antibiotic and an extract treatment (*p* > 0.05). However, at high concentrations, a reduction in viability was shown, most likely as a result of the extract and gentamicin’s additive effects. These findings were then contrasted with the literature’s data. Thus, to the best of our knowledge, there has been only one previously published study regarding the cytoprotective effect of *Cornus mas* L. fruit extract in renal cell injury in vitro. Thus, Yarim et al. [34] demonstrated the cytoprotective effect of these fruits against cisplatin-induced nephrotoxicity by MTT assay. Cell viability in the cisplatin-treated group was 42%, while cisplatin and *Cornus mas* L. extract treated cells registered a significantly higher percentage of cell viability, i.e., 59%. The results of our investigation are generally consistent with this previous publication. 

The findings of the Annexin-V FITC test were comparable to those of the MTT assay. Thus, the gentamicin-treated cells showed a significantly lower percentage of viability when compared to control (*p* < 0.0001). This group additionally contained the highest proportion of cells in the necrotic phase, emphasizing the adverse effects of gentamicin on renal epithelial cells. Likewise, extract-treated cells had a significantly decreased percentage of vitality in comparison to control (*p* < 0.0001). On the contrary, when compared to cells that had just suffered gentamicin treatment, a higher percentage of cells were still viable. The greatest concentration from the extract-treated cells obtained the highest number of necrotic cells, outlining the information mentioned above regarding the pro-oxidant properties of polyphenols at high levels. Furthermore, there was no discernible loss in cell vitality at the first two concentrations in the group that received gentamicin and *Cornus mas* L. extract together, when compared to cells treated with extract only (*p* > 0.05). The last concentration, however, was found to be the most cytotoxic, reporting a lower percentage of viability than cells that were treated with the extract (*p* < 0.05) or even gentamicin (*p* < 0.05). This discovery might have relied on the extract and gentamicin’s additive pro-oxidant effects. 

The present study offers originality and, to the best of our knowledge, the first data regarding the apoptotic measurements of the tested ethanolic extract from *Cornus mas* L. Consequently, our study was contrasted to the protective effects of other natural substances in gentamicin-induced nephrotoxicity. As such, epigallocatechin gallate (EG), an active constituent of green tea, was studied by Yue et al. [52] for its effects on renal cells during gentamicin stress. The assessment of the cellular apoptotic rate by flow cytometry was performed. This indicated that the gentamicin-treated cell group had a substantially increased number of apoptotic cells. The use of EG as a therapeutic agent prevented these outcomes, with the rate of cell apoptosis reducing in the group receiving treatment with both gentamicin and the natural substance. These results are mostly comparable to those of our investigation, with the exception of the last concentration, which recorded noticeably higher values than the gentamicin group in both early and tardive apoptosis. In another study, sulforaphane (SFN), a naturally occurring isothiocyanate present in crucifers, was examined in order to determine whether it could protect LLC-PK1 cells from acute renal injury induced by gentamicin. In comparison to untreated cells, gentamicin treatment resulted in a 1.5-fold increase in necrotic cells and a 4.8-fold increase in apoptotic cells, aspects revealed by the flow-cytometry analysis. After receiving SFN therapy, the rise in apoptosis was greatly reduced. The rate of cell necrosis, however, did not significantly decrease after this natural compound was applied [53]. In our current study, a significant increase in cell necrosis compared to cell apoptosis was noted, probably due to the instability of the primary renal cell culture. Moreover, the cell groups treated with extract + antibiotic had a lower percentage of necrotic cells compared to the cells treated only with antibiotic. Shin et al. [54] investigated the nephroprotective effects of red ginseng extract (RGE) in acute renal injury induced by gentamicin on NRK-52E cells. The flow-cytometric analysis revealed that the nephrotoxic induced a significant increase in early apoptosis (47.6%) when compared to the control group (2.7%), with RGE improving these aspects. The increase in early apoptosis was even higher than the increase in cell necrosis in our study (47.6% vs. 30.87%). This finding may be the result of the different gentamicin concentrations and cell lines employed in each study. 

Therefore, it could be emphasized that, by displaying a more thorough cytotoxic activity utilizing the MTT test and Annexin V-FITC assay as well, this is the first article revealing that *Cornus mas* L. extract exhibits cytoprotective effects on primary renal cells stressed with gentamicin. Future interest in minimizing kidney injury may result from this study.

## 4. Materials and Methods

### 4.1. Chemicals and Reagents 

Sigma-Aldrich provided phenolic compounds and carotenoid pigments standards (Darmstadt, Germany). All tested samples were purified via a 0.45 µm MF-Millipore™ Membrane Filter from Merck prior to HPLC Analysis (Darmstadt, Germany). Plant Flavonoids Colorimetric Assay Kit and Annexin V-FITC with propidium iodide (PI) flow-cytometry Kit were both acquired from Elabscience Biotechnology Inc. (Houston, TX, USA) and Thermo Fisher Scientific Inc. (Waltham, MA, USA), respectively. Bacterial reference strains were obtained from Oxoid Ltd. (Hampshire, UK) and culture mediums, such as Mueller Hinton Broth and Mueller Hinton agar, were purchased from Merck (Darmstadt, Germany). Enzymatic mixing solutions for cell cultures were acquired from Sigma-Aldrich (St. Louis, MO, USA), while the substances for the culture medium were purchased from Gibco Life Technologies, Paisley, UK.

### 4.2. Phytochemical Profile of Cornus mas L. Fruit Extracts

#### 4.2.1. Harvesting Fruit Samples

Cornelian cherry twigs, containing leaves and flowers, and fruits were collected from shrubs growing in natural habitats in the steep hills of Mărişel commune, Cluj county, Romania (46°40’03.7″ N 23°06’35.6″ E). The fruits were harvested between August and September 2020. All the plant’s components were identified and verified by Dr. Andrei Stoie (Professor of Botany, Faculty of Agriculture, University of Agricultural Sciences and Veterinary Medicine, Cluj-Napoca, Romania). A voucher specimen was deposited in the Botanical Department’s herbarium, Faculty of Agriculture, University of Agricultural Sciences and Veterinary Medicine, Cluj-Napoca, Romania. After the fruits were harvested and identified, the twigs, leaves and stones were manually removed. The entire quantity of fruit pulp was then portioned into 100 g sample pouches and preserved at −18 °C in a refrigerator until further proceedings. 

#### 4.2.2. Preparation of Extracts

The fruits had been thawed and dehydrated using a TEESA TSA3031 dehydrator at a temperature of 45 °C for 7 days. After dehydration, they were pulverized into powder. The extraction was performed on 10 g of fruit powder using 100 mL of 96% ethanol. The resulting mixture had been subjected to homogenization for 2 h, with the help of a magnetic stirrer, at 1000 rpm. The mixture was then filtered, using a funnel and cotton wool, and the residue was re-extracted using the same procedures. The resulting extracts were pooled and concentrated by evaporation, using an Eppendorf Concentrator Plus evaporator at 45 °C. Lastly, the volume obtained was measured and utilized for direct determinations, namely total phenolic content, total flavonoids, LC-MS analysis, antioxidant capacity, antimicrobial activity and cytotoxic assays. All phases of extraction were performed in triplicate. 

In parallel, an ethyl ether re-extraction was performed in order to determine the fat-soluble compounds, particularly total carotenoids. As such, water, ethyl ether and the ethanolic extract were poured into a separatory funnel. Two phases were observed: the upper organic phase and the lower aqueous phase. The organic phase was separated and passed through anhydrous Na_2_SO_4_ to remove traces of water and was later subjected to rotary evaporation at 40 °C. As a final extraction phase, the oleoresin residuum was resuspended in a known volume of ethyl ether for subsequent assessments.

#### 4.2.3. Total Phenolic Content Assay

The Folin–Ciocalteu method was employed to assess total polyphenolic concentration. This method was performed according to Moldovan et al. [45] with slight modifications. A calibration curve of gallic acid was obtained, with eight different concentrations, ranging from 50 to 450 µg/mL. At 765 nm, the absorbance was measured using a microplate spectrophotometer (SPECTROstar^®^ Nano—BMG Labtech, Ortenberg, Baden-Württemberg, Germany). The values were presented in mg of GAE (gallic acid equivalent) per mL of extract. The measurements were conducted in three replicates [55].

#### 4.2.4. Total Flavonoid Content Assay

The total flavonoid content was assessed using a colorimetric assay kit (Plant Flavonoids Colorimetric Assay Kit—Elabscience Biotechnology Inc., Houston, TX, USA). The principle of assessment involves the reaction of flavonoids and aluminum ions in a basic medium, which leads to the formation of a red complex. The samples were prepared according to the instructions on the kit. The absorbance of the samples was determined in contrast with a blank sample (bidistilled water) at 510 nm, using the same microplate spectrophotometer as mentioned in the previous determination. A standard curve was obtained for calculation by diluting the standard solution of 1 mg/mL with bidistilled water to six different concentration levels, between 20 and 150 µg/mL. The outcomes were given in µg per mL of extract. The analyses were performed in triplicate [55].

#### 4.2.5. HPLC-DAD-ESI-MS Screening

Chromatographic separation of phenolic compounds in the ethanolic extract of *Cornus mas* L. fruits was conducted on an Agilent 1200 HPLC system packed with photodiode UV-Vis detector (DAD) coupled to a single mass detector (MS), model 1160 (Agilent Technologies, CA, USA). The protocol for separating the compounds was carried out in accordance with Dumitraş et al. [55], who detailed the methodology. Three calibration curves were created for quantification of the phenolic compounds, by injecting five different concentrations of 99% purity standard substances as follows: chlorogenic acid for phenolic acids, rutin for flavonoids and cyanidin for anthocyanins. The calculation was made using a mathematical formula: y = 40.7 × (−71.5) (R2 = 0.9995). Spectral values had been recorded in the range of 200–600 nm, with phenolic acids being detected at 280 nm, flavonoids at 340 nm and anthocyanins at 520 nm. For LC-MS, the following working conditions were employed: capillary voltage of 3000 V, temperature of 35 °C, nitrogen flow of 7 L/min and monitoring range of 100–1200 m/z. Agilent ChemStation software was used to collect data and interpret results. 

#### 4.2.6. Total Carotenoid Content Assay

In accordance with Dumitraş et al. [55], a UV spectrophotometric method was assessed to determine the concentration of total carotenoids. The extract’s absorption spectrum was measured between 350 and 700 nm using the same microplate spectrophotometer mentioned above. The quantity of carotenoid pigments was calculated [56] and the results were expressed as µg/g of DM (dry mass). The tests were conducted in three replicates.

### 4.3. Antioxidant Capacity

#### 4.3.1. Radical Scavenging Activity Assay (DPPH)

The principle of DPPH method is based on the gradual formation of a colorless compound as the intensity of the purple color of the DHPP solution decreases after the addition of an antioxidant. Thus, by monitoring the decrease in DPPH absorbance, the antioxidant interest of the compound can be determined [57]. The assay was performed in accordance with Dumitraş et al. [55], with slight modifications. In brief, 2 mL of 0.1 g/L DPPH solution in methanol was mixed with 2 mL of extract in various quantities. The samples were kept in a 40 °C thermic bath for half an hour before measuring the change in absorbance at 517 nm. The following percentage of DPPH scavenging capacity was estimated as follows: DPPH inactivation capacity % = (AC − AS/AC) × 100, where AC represents control absorbance and AS represents sample absorbance. The results were provided in IC_50_ (mg/mL). 

#### 4.3.2. Ferric Reducing Antioxidant Power Assay (FRAP)

The FRAP method was implemented following the methodology described by the study mentioned earlier [55], with minor adjustments. The assay’s principle is built on antioxidants’ ability to convert Fe^3+^ to Fe^2+^, an ion that subsequently forms blue complexes in the presence of TPTZ (2,4,6-Tri (2-pyridyl)-s-triazine) and can be detected at 593 nm [58]. Firstly, FRAP reagent was prepared using a known volume of TPTZ solution, ferric chloride solution and acetate buffer. Following this phase, 6 mL of FRAP reagent was mixed with 0.8 mL of deionized water in 4 mL of extract. Correspondingly, a blank solution was created by substituting water for the sample. Measurements of absorbance at 593 nm were used to correlate color change with antioxidant capacity. The antioxidant activity was calculated using a Trolox calibration curve (R2 = 0.992). The scores were expressed as μmol Trolox equivalents/mL of extract.

### 4.4. Antimicrobial Activity

The antimicrobial properties of *Cornus mas* L. extract were in vitro evaluated using the agar well diffusion assay, being an adapted EUCAST (European Committee on Antimicrobial Susceptibility Testing) disk-diffusion assay [59]. Six bacterial reference strains obtained from Oxoid Ltd. (Hampshire, UK) were integrated, namely *Staphylococcus aureus* ATCC 25923 (methicillin-susceptible *S. aureus*, MSSA), *Staphylococcus aureus* ATCC 700699 (methicillin-resistant *S. aureus*, MRSA), *Bacillus cereus* ATCC 14579, *Enterococcus faecalis* ATCC 29219, *Escherichia coli* ATCC 25922 and *Pseudomonas aeruginosa* ATCC 27853. Each bacterium was cultured on Mueller Hinton (MH) broth and agar, mediums that were purchased from Merck (Darmstadt, Germany, catalogue number 70192 and 70191-500G). A 24 h pure culture was used to prepare the bacterial inoculums by suspending colonies in sterile saline to obtain 10E6 colony forming unit (CFU)/mL, according to the McFarland scale. After inoculums were “flood-inoculated” on MH agar plates, 6-millimeter diameter wells (three for each testing) were aseptically made into the MH agar and filled with 60 μL of extract. Both negative (70% ethanol in water *v*/*v*) and positive controls (standard antibiotics disks: gentamicin (10 µg), amoxicillin–clavulanic acid (20–10 µg) from Oxoid Ltd., Hampshire, UK, catalog number CT0794B and CT0223B) were included. Following 24 h of incubation at 37 °C, the values of growth inhibition zone diameters (in mm) were measured. A second method, namely the broth microdilution method, was utilized to establish the minimum inhibitory (MIC) and bactericidal (MBC) concentrations. Briefly, two-fold serial dilutions of the tested extract in MH broth were made in 96-well bottom “U” polystyrene plates. The resulting concentrations (ranging from 650 μg GAE/μL to 5.07 μg GAE/μL) were cultured with 5.0 µL bacterial inoculum for 24 h at 37 °C. Reading of MICs values took into account the lowest concentrations able to inhibit the visible bacterial growth (no turbidity in the well) compared to the negative control (MH broth). MBC values were established following the 24 h culture on MH agar of 10.0 µL from each well and considering the lowest concentrations associated with no visible bacterial growth on the agar plates. MH broth was also included as MIC negative control. In addition, the MIC index calculated based on the ratio MBC/MIC indicated whether the extract displays a bactericidal (MBC/MIC ≤ 4) or bacteriostatic (MBC/MIC > 4) effect against the tested bacterial strains [60,61].

### 4.5. Cell Cultures and Cytotoxic Activity

#### 4.5.1. Experimental Animals and Protocols

The experiment was conducted in the authorized Animal Research Facility of the Faculty of Veterinary Medicine, Cluj-Napoca, Romania. The Institute of Oncology “Prof. Dr. Ion Chiricuţă”, Cluj-Napoca, Romania provided one adult, 6 days pregnant female C57BL/6J mouse, weighing 25 g, which was used in the experiment. In accordance with ISO 10993-6 requirements [62] and Regulation 63/2010/EU, the female was maintained in an aerated cage, at a temperature of 25 ± 2 °C and a relative humidity of 55% ± 10%, with a 12 h diurnal period. In addition, the individual had unrestricted access to fresh water and regular food for rodents, provided by the Cantacuzino Institute, Bucharest, Romania. Before the experimental procedures, the individual was acclaimed in this environment for 1 week. All animal operations adhered to the standards of Regulation 63/2010 and state legislation no. 43/2014. The study was authorized by the Ethics Committee of the University of Agricultural Sciences and Veterinary Medicine, Cluj-Napoca, Romania (no. 256/21.04.2021) and by the Regional Sanitary Veterinary and Food Safety Authority (no. 274/12.11.2021). Surgical techniques were carried out in accordance with ISO 10993-6. 

At the end of the accommodation period, the female was mercifully euthanized under general anesthesia through cervical dislocation, on day 13 of pregnancy. After this procedure, incisions in the abdominal cavity and further in the uterus were performed, with the extraction of five individual fetuses that were separated and washed in Dulbecco’s phosphate buffer saline (PBS). Furthermore, the fetuses’ abdominal cavities were incised and the kidneys were collected and added to PBS solution for further proceedings.

#### 4.5.2. Renal Epithelial Cell Cultures

In order to obtain primary renal cultures, cells were isolated from the kidneys of mouse fetuses through a mixed method which implies tissue explants and enzymatic treatment. The renal capsule was removed, and kidneys were finely minced and further treated for 30 min with an enzymatic mixing solution consisting of collagenase IV 0.1% and 0.1 mg/mL Dispase (Sigma-Aldrich, Saint Louis, MO, USA). Cell suspension together with residual tissue explants was added to culture plates that had been pretreated with 4% porcine gelatin (Sigma-Aldrich, Saint Louis, MO, USA). For propagation, Dulbecco’s Modified Eagle Medium was used, as it was supplemented with 10% fetal bovine serum, 100 mg/mL streptomycin, 100 U/mL penicillin and 50 mg/mL gentamicin (substances purchased from Gibco Life Technologies, Paisley, UK). Furthermore, the cultures had been incubated at 37 °C, in a microclimate enriched with 5% CO_2_ and 90% humidity for 5 days. Additionally, the cultures were washed with PBS solution and treated for 10 min at 37 °C with Trypsin–EDTA solution (Sigma-Aldrich, Saint Louis, MO, USA), after which the cell suspension was centrifuged at 1200 rpm for 7 min. Subculturing was completed at a concentration of 5 × 10^3^ cells/plate.

#### 4.5.3. Cell Viability Assay

The MTT (3-(4,5-dimethylthiazol-2-yl)-2,5-diphenyltetrazolium bromide) colorimetric assay was used to assess the extract’s potential cytotoxicity. The method functions by reducing the yellow MTT compound to purple formazan crystals using enzymes from metabolically active cells. The formazan is then dissolved in 100% isopropanol, and its concentration is measured spectrophotometrically at 450 nm. The protocol was carried out in accordance with previous research with slight modifications [48]. Therefore, a concentration of 1 × 10^4^ cells/well, corresponding to 200 µL cell suspension, was obtained. The cell suspension was placed in each well of 96-well plates containing normal propagation medium and was further held in abeyance for 24 h. Following that, the extract to be evaluated was added to wells in volumes of 10, 15 and 20 μL, in three differing concentrations, namely CM1, CM2 and CM3. The amount of polyphenolic compounds found in the fruit extract was used to calculate these concentrations. Thus, CM1 denotes 130 μg GAE/μL polyphenols, CM2 denotes 195 μg GAE/μL polyphenols and CM3 denotes 260 μg GAE/μL polyphenols. Several cell cultures were treated with 100 mg/mL gentamicin in three different concentrations (G1 = 100 μg/μL, G2 = 150 μg/μL and G3 = 200 μg/μL), while several others were treated with *Cornus mas* L. extract and gentamicin together. Untreated cells were used as negative control, while gentamicin-treated cells were used as positive control. Cell proliferation was assessed after 24 h by removing the culture medium and adding 100 μL of MTT solution (1 mg/mL) (Sigma-Aldrich, St. Louis, MO, USA). The MTT solution was removed from each well after 3 h of incubation at 37 °C in darkness, and 150 μL of DMSO (dimethyl sulfoxide) solution was added. The BioTek Synergy 2 spectrophotometer (Winooski, VT, USA) was used to measure the intensity of the chromogenic reaction at 450 nm. The percentage of cell viability was calculated based on a mathematical formula: Viability% = AT/AC × 100, where AT represents the absorbance of treated cells and AC represents the absorbance of control cells. All tests were run in three replicates.

#### 4.5.4. Cell Apoptotic Assay

The Annexin V-FITC cell apoptosis detection kit (Thermo Fisher Scientific Inc., Waltham, MA, USA) was used to determine the apoptosis rate of renal epithelial cells. This determination was assessed following the guidelines represented in the kit. A total of 2.5 × 10^5^ cells/well were planted in 12-well plates and further treated with the same concentrations of extract (CM1–CM3), gentamicin and a combination of both. Furthermore, PBS (phosphate bovine serum) solution was used to wash the cells before resuspension. To resuspend the cells, 500 μL of Annexin V Binding Buffer was introduced in each well. Subsequently, a total of 5 μL of Annexin V-FITC reagent and 5 μL of fluorescent substance (100 g/mL PI—propidium iodide) were added. Following these procedures, the samples were vortexed and incubated in a sunless environment at room temperature for 15 min. At the end of the incubation period, the samples were analyzed by flow cytometry using the FACS technique (fluorescent sorting of activated cells). As such, flow cytometry was carried out using a flow cytometer outfitted with a 488 nm, air-cooled, 20 mW solid-state excitation laser, as well as a 530/30 FITC filter and a 575/26 PI filter for fluorescence detection. The data were analyzed using the FACSDiva 6.1.2 software (Becton Dickinson, San Jose, CA, USA). As for interpretation, it was assumed that Annexin V-FITC+ and PI− indicate apoptosis in its early stages, meanwhile Annexin V-FITC+ and PI+ indicate tardive apoptosis. Likewise, Annexin V-FITC− and PI+ suggest cells to be necrotic and Annexin V-FITC− and PI− suggest cells to be viable [55]. 

### 4.6. Statistical Analysis 

The results were expressed as mean ± standard deviation (SD) after each assessment was conducted in triplicate. The statistical software Graph Pad Prism 9 (San Diego, CA, USA) was used to perform the statistical analysis. Utilizing one-way ANOVA and *t*-test functions, data analysis was completed. The significance level was established at *p* < 0.05.

## 5. Conclusions

To the best of our knowledge, this is the first paper that analyzes the extract of *Cornus mas* L. fruits in relation to its phytochemical profile, antioxidant capacity, antimicrobial potential and cytoprotective effects. 

*Cornus mas* L. fruits have been shown to have high concentrations of biologically active substances, particularly phenolic compounds, whereas carotenoids were only identified in relatively low concentrations when compared to other plant components. The fruit extract demonstrated a significant in vitro antioxidant capacity and antimicrobial activity against all tested reference strains. Furthermore, with the exception of the high dose, which had a similar nephrotoxic impact to that of gentamicin, it also demonstrated cytoprotective benefits in gentamicin-induced nephrotoxicity on mice renal epithelial cells. 

Without intending to minimize the significance of this paper, additional research is necessary to elucidate the underlying physiological mechanisms of the cytoprotective effects of *Cornus mas* L. fruits in gentamicin-induced nephrotoxicity and establish the toxic dose of the extract. Additionally, this work can serve as a foundation for future investigations into the antimicrobial potential of *Cornus mas* L. fruits in pathogens involved in diseases of the urinary system in both humans and animals. 

## Figures and Tables

**Table 1:** Total phenolic content, flavonoids and carotenoids of *Cornus mas* L. fruit extracts.

Fruit Extract Samples	Total Polyphenols (TP) * (mg GAE/mL)	Total Flavonoids (TF) * (µg/mL)	Total Carotenoids (TC) ** (µg/g DM)
S1	0.872	139.17	3.8
S2	0.869	141.23	4
S3	0.876	137.03	3.6
**Mean ± SD**	**0.872 ± 0.0035**	**139.14 ± 2.100**	**3.8 ± 0.0002**

S1–S3 = analyzed fruit extract samples; * Ethanolic extracts (for TP and TF measurements); ** Ethyl Ether re-extraction of the initial Ethanolic extracts (for TC measurements); mean ± SD represents the results of the triplicate measurements; DM = dry mass.

**Table 2:** HPLC-DAD-ESI-MS analysis of phenolic compounds of *C. mas* L. extract.

Nr. Peak	Retention Time R_t_ (min)	UV λ_max_ (nm)	[M + H] ^+^ (m/z)	Compound	Subclass	Concentration (μg/mL)
1	3.28	270	333, *171*	Gallic acid glucoside	Hydroxybenzoic acid	248.516
2	3.82	520, 322, 280	595, *449*	Cy3-O-(coumaroyl-glucoside)	Anthocyanin	6.439
3	11.34	340, 290	391	Loganin	Iridoid	111.478
4	11.86	520, 280	449, 595	Cy 3-O-galactoside Cy 3-O-robinobioside	Anthocyanin	89.484
5	12.22	330	355, *163*	5-Caffeoylquinic acid (Chlorogenic acid)	Hydroxycinnamic acid	23.189
6	12.59	514, 280	433, 579	Pg 3-O-galactoside Pg 3-O-robinobioside	Anthocyanin	42.826
7	13.04	322	181	Caffeic acid	Hydroxycinnamic acid	57.150
8	14.29	350, 280	359	Sweroside	Iridoid	31.602
9	15.99	360, 255	611	Q 3-O-rutinoside (Rutin)	Flavonol	29.635
10	17.21	340, 261	449	K 3-O-galactoside	Flavonol	11.023
11	17.49	280	579, *291*	Procyanidin dimer	Flavonol	195.826

Triplicate results were displayed as mean and presented in µg/mL of extract (Cy = Cyanidin, Pg = Pelargonidin, Q = Quercetin, K = Kaempferol).

**Table 3:** In vitro antibacterial activity of *Cornus mas* L. extract by well diffusion method.

Sample	Zone of Inhibitions (mm)					
	MSSA *	MRSA *	*Bacillus cereus*	*Enterococcus faecalis*	*Escherichia coli*	*Pseudomonas aeruginosa*
*Cornus mas* L.	20 ± 0.00 ^a^	16.33 ± 0.47 ^a^	17 ± 0.00 ^a,b^	22.33 ± 0.47 ^a^	20 ± 0.00	22.33 ± 0.47
Amoxicillin–clavulanic acid	29 ± 0.00	28 ± 0.00	20 ± 0.00	17 ± 0.00	19 ± 0.00	0
Gentamicin	20 ± 0.00	17 ± 0.00	26 ± 0.00	0	19 ± 0.00	19 ± 0.00

MSSA, methicillin-susceptible Staphylococcus aureus; * MRSA, methicillin-resistant *Staphylococcus aureus*; values represent the mean ± SD of two independent measurements. ^a,b^ Means with different subscript letters within a row are significantly different at *p* < 0.05 (unpaired *t*-test); antibiotic disks: amoxicillin–clavulanic acid (20–10 µg), gentamicin (10 µg).

**Table 4:** In vitro antibacterial activity of *Cornus mas* L. extract by broth microdilution assay.

MIC Index MBC (µg GAE/µL)/MIC (µg GAE/µL)						
**Sample**	MSSA	MRSA	*Bacillus* *cereus*	*Enterococcus faecalis*	*Escherichia coli*	*Pseudomonas aeruginosa*
***Cornus mas* L.**	**1** 5.07/5.07	**1** 5.07/5.07	**1** 5.07/5.07	**2** 10.1/5.07	**2** 10.1/5.07	**1** 81.2/81.2

## Data Availability

The data presented in the study are available in the article.
